# A global dataset for assessing nitrogen-related plant traits using drone imagery in major field crop species

**DOI:** 10.1038/s41597-024-03357-2

**Published:** 2024-06-05

**Authors:** Diogo Castilho, Danilo Tedesco, Carlos Hernandez, Beata Emoke Madari, Ignacio Ciampitti

**Affiliations:** 1 Graduate Program in Agronomy, Federal University of Goiás, Goiânia, Goiás Brazil; 2 Brazilian Agricultural Research Corporation (Embrapa Rice and Beans), Santo Antônio de Goiás, Goiás Brazil; 3 Department of Agronomy, Kansas State University, 1712 Claflin Rd., Manhattan, KS 66506 USA

**Keywords:** Leaf development, Plant sciences, Light responses

## Abstract

Enhancing rapid phenotyping for key plant traits, such as biomass and nitrogen content, is critical for effectively monitoring crop growth and maximizing yield. Studies have explored the relationship between vegetation indices (VIs) and plant traits using drone imagery. However, there is a gap in the literature regarding data availability, accessible datasets. Based on this context, we conducted a systematic review to retrieve relevant data worldwide on the state of the art in drone-based plant trait assessment. The final dataset consists of 41 peer-reviewed papers with 11,189 observations for 11 major crop species distributed across 13 countries. It focuses on the association of plant traits with VIs at different growth/phenological stages. This dataset provides foundational knowledge on the key VIs to focus for phenotyping key plant traits. In addition, future updates to this dataset may include new open datasets. Our goal is to continually update this dataset, encourage collaboration and data inclusion, and thereby facilitate a more rapid advance of phenotyping for critical plant traits to increase yield gains over time.

## Background & Summary

Agriculture is an important industry, serving as the foundation of food security and of the global economy^1^. The complexity of biological systems is reflected in the spatial temporal variability of the soil and crop N status within a field^2^. To ensure optimal use of outputs, fertilizers should be provided at the right time, place, with an adequate source and at the right rate, only when necessary^3^. Therefore, a variable nitrogen (N) management strategy must be implemented to optimize fertilizer N rates, economic benefits, and maintaining or increasing both yield and quality^4^.

N is a critical element for crop growth and one of the most important nutrients in agriculture to improve crop yield and for protein formation^5^. Furthermore, the utilization of the right fertilizer N rate is crucial not only to increase yields but to reduce the environmental footprint of this practice^3,6^. Traditional methods for detecting crop N status involve time-consuming field sampling and costly laboratory analysis^7^. Monitoring crop N status efficiently and effectively remains an urgent problem to be solved^8,9^.

In recent years, technological innovations based on the utilization of multispectral and hyperspectral sensors mounted in different platforms help to provide critical imagery data for phenotyping and developing new tools for precision agriculture^10^. The emergence of unmanned aerial vehicles (UAV, or commonly known as drone) has advanced remote sensing applications at fine scales. UAV have gained significant scientific and public interest, due to their flexibility, easiness to use, and affordability^11,12^. The aerial platform and sensor cost with a rapid image availability make this equipment valuable for assessing critical plant traits for advancing yield gains^13,14^.

Characterization of key plant traits can vary depending on the crop and growth stage. Many efforts have been dedicated to identifying VIs that best correlates to plant traits^15–18^. The most relevant linked to crop N status include leaf N content, leaf N concentration (LNC), plant N concentration (PNC), N nutrition index (NNI), and N concentration (NC) for different plant fractions^19–24^. Several crops have been investigated using drone technology to assess plant traits, including but not limited to wheat (*Triticum aestivum* L.)^15^, corn (*Zea mays* L.)^16^, rice (*Oryza sativa* L.)^17^, and barley *(Hordeum vulgare*)^18^. Different crops may require species-specific VIs to better characterize crop N status, as differences in leaf structure, canopy architecture, N allocation, and phenological stage should be taken into account when comparing across them^4,25,26^. In addition, other factors such as soil exposure, crop residues, and N application levels can also affect the stability of an index^27^, provide restrictions to use a more universal index for accurately estimate similar plant traits across crop species^11^. For example, NDVI has been found to be a reliable index for N estimation in corn but less effective for rice^28–31^. Therefore, it is important to identify and evaluate the most effective VIs more directly targeting specific plant traits across major field crops.

A critical challenge as technology is evolving and the number of published studies on this topic grows exponentially with time is to keep up with the current progress and identify research knowledge gaps. Furthermore, as the analysis is based on a single experiment with N treatments and small plots, further research is needed to translate current findings to real-world scenarios^32^. Most studies using UAV assess nutrient content using a simple regression model, typically linear models^14^, and usually focused on a few plant traits. To date, the existing academic literature on the merger of studies utilizing the same VIs and plant traits is limited. Therefore, developing a more organized and structured review can help identify promising VIs and plant traits while developing an open dataset to assist future progress on this topic.

It is acknowledged that variances in plant traits accuracies exist between studies^33,34^. These discrepancies often arise from differences such as geographical location, types of drones and camera sensors, and the application of signal processing techniques (multivariate linear methods, (e.g., partial least squares regression, stepwise multiple linear regression, and multiple linear regression), multivariate non-linear methods (e.g., random forest and support vector machine), and univariate methods (e.g. linear regression). As most studies using UAV assess nutrient content using univariate methods^14^, typically linear or non-linear regressions, we focused our study on gathering information on studies that used VIs to predict any N-related trait and/or yield.

Following this rationale, a systematic review process focusing on retrieving datasets on the state of the art in drone-based plant traits assessment was executed. Our global dataset focuses on major field crop species, 11 total, retrieved from studies published during the last two decades (2000 to 2023) in 13 countries. The final dataset contains 41 peer-reviewed scientific manuscripts focusing on the relationship between VIs and plant traits for characterizing crop N status and identifying knowledge gaps to guide future research on drone-based plant traits assessments.

## Methods

**Fig. 1:**
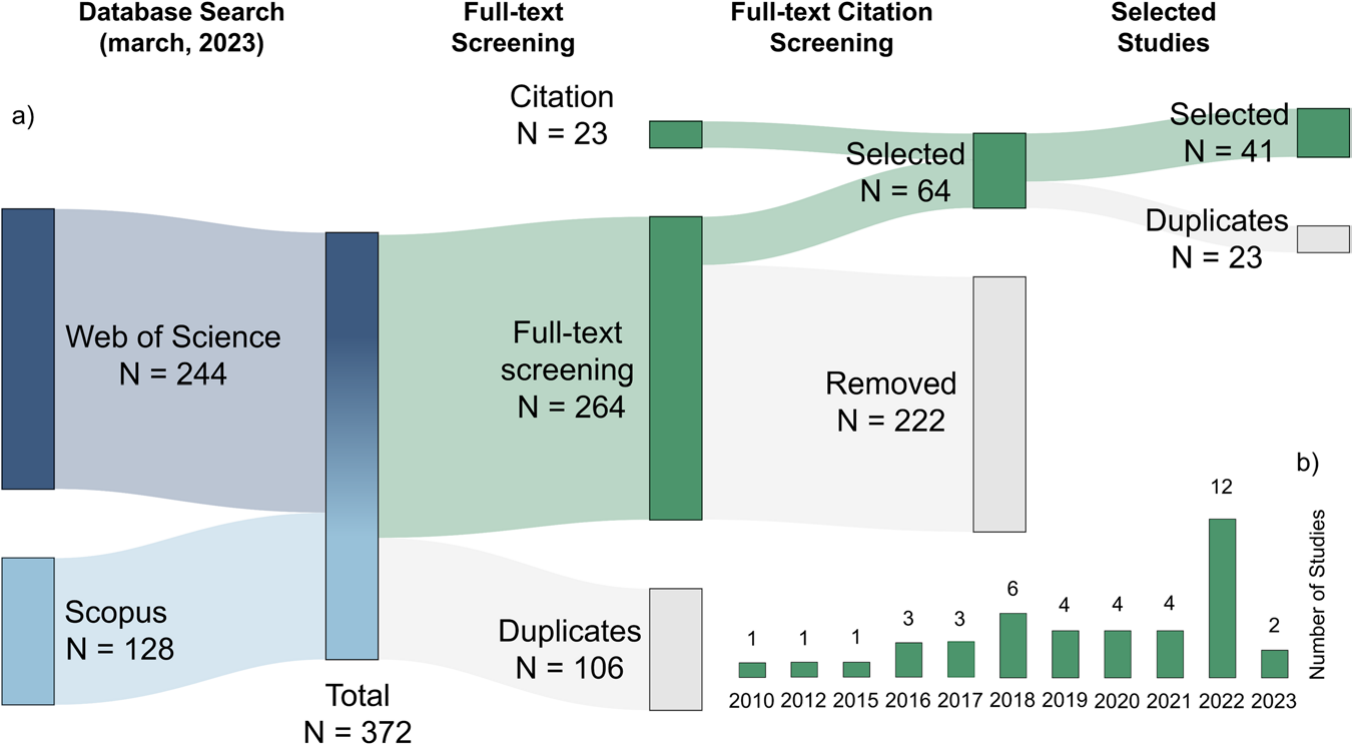
(**a**) Sankey diagram illustrating the studies search, collection, filtering, and selection. (**b**) Number of studies selected per year.

A literature search was conducted, involving identification, screening, eligibility, and inclusion of relevant records (Fig. 1a). The Scopus and Web of Science search engines were the main data sources. The keywords “multispectral airborne images” or “drone” or “UAV” or “UAS” or “unmanned aerial vehicle” or “remotely piloted aircraft system”, AND “nitrogen” AND “yield” were included in the search criteria, restricting the duration from 2000 to March 2023 to identify the most promising modern technologies.

After retrieving all relevant records (number of studies, n = 372), a first screening process was performed to remove duplicates (n = 264) for further processing. As a next step, an intensive analysis/full text reading was executed. Studies presenting the following criteria were excluded of the final dataset: 1) languages other than English, 2) unavailability of full-text publication, 3) lack of focus on field experiments (other setting greenhouse, growth chamber, pots, etc.), 4) articles that did not use drones to collect RGB, multi- or hyperspectral images, 5) used more than one crop/plant mixed (not focus on a single cropping specie), 6) study not focused on plant N/yield association, 7) lack of observed N determinations (excluding indirect N measurements such as chlorophyll meters, handheld sensors), and lastly, 8) those studies only benchmarking UAV derived imagery data with handheld sensors.

In the next step, full-text screening was performed to exclude studies that did not report data on VIs and plant trait, removing 222 papers. An additional 23 papers were identified and reviewed by examining citations from the remaining manuscripts, resulting in the inclusion of 22 additional studies. These studies were checked for duplicates. As a result, a final database comprises 41 articles published between 2010 to 2023 (Fig. 1b).

A total of 41 records were identified fulfilling the main criterion of crop N estimation using different crops with RGB, multispectral, and hyperspectral data derived from the utilization of drones. The data retrieved from each paper included: i) geographic location of the experiments, ii) crop species, iii) plant traits (N content/concentration, N nutrition index (NNI), N uptake, leaf/plant N accumulation, canopy N content, N rate, and biomass), iv) VIs/bands, v) applied N rates, and vi) timing of UAV flights/phenological stages (further details presented in Table 1).

**Table 1:** Study identification (ID), country, crop, phenological stage/time, plant traits and vegetation index.

ID	Country	Crop	Growth Stage/Time^1^	Plant Traits	Vegetation Index^2^	Ref
01	Spain	Wheat	Stem Elongation, Flowering	Yield	NDVI	^15^
02	Switzerland	Winter Wheat	Tillering, Stem Elongation, Heading	PNC, NNI, N uptake	MCARI/MTVI2, NDRE	^40^
03	Australia	Cotton	First Flower, First Cracked Boll, Maturity	PNC, N uptake	SCCCI, TCARI/OSAVI, TGI, VARI, NDRE, NDVI	^41^
04	Italy	Winter Wheat	Stem Elongation, Heading	Yield	NDVI	^3^
05	USA	Corn	VT, R3, R4, R5, R6	Yield, LNC	CIg	^16^
06	Italy	Bermudagrass, Tall fescue	Mature	LNC	DGCI	^46^
07	China	Winter Wheat	Stem Elongation	NNI, Relative Yield	—	^47^
08	Brazil	Soybean	R3	Leaf N Content	CVI, GRVI, RECI, SCCCI	^48^
09	Italy	Corn	V7	Yield	NDRE	^49^
10	China	Winter Wheat	Stem Elongation, Heading, Flowering	PNC, LNC, LND, PND	CIVE, ExR, GLI	^34^
11	China	Wheat	Stem Elongation - Heading - Flowering - Ripening	LNC	NDRE	^50^
12	Germany	Winter Wheat	Stem Elongation, Heading, Flowering, Ripening	PNC, Yield	REIP	^32^
13	Germany	Wheat	Stem Elongation, Heading, Flowering	N uptake	REIP	^51^
14	China	Winter Wheat	Tillering, Heading	NNI, Yield	—	^52^
15	Spain	Barley	150 DAP^3^	Yield	NDVI, OSAVI, RDVI, SAVI, WBI	^18^
16	Reunion Island	Sugarcane	Grand Growth	CNC, Leaf N Content	NDVI, GNDVI, SRPI	^12^
18	China	Winter Wheat	Tillering - Heading	PNC	NDI (365, 410), SR (787/765)	^53^
19	China	Rice	Filling	LNC	DGCI	^17^
17	USA	Sorghum	120 DAP^3^	Biomass	RDVI	^54^
20	China	Oilseed Rape	Vegetative	NNI	CIRE, VARI	^55^
21	China	Rice	Booting, Heading	Yield	SAVI, WDRVI	^56^
22	Spain	Corn	V12	N rate	NDVI, GRVI, WDRVI	^57^
23	USA	*Miscanthus × giganteus*	Mid-summer growing season	Biomass	NDRE	^58^
24	Brazil	Corn	V12	LNC	GNDVI, NDRE, NDVI, NIR, Red, SAVI	^59^
25	Thailand	Corn	Vegetative, Reproductive	Yield, Biomass	NDRE, NDVI	^42^
26	USA	Corn	R1	LNC	VEG	^60^
27	Zimbabwe	Corn	R1	LNC	NDVI	^61^
28	Finland	Grass Swards	06, 15, 19, 28/June	Biomass	MSAVI	^62^
29	USA	Spring Wheat	Tillering, Heading	PNC	CIg, CIRE, EVI2, MTCI, NDRE, NDVI	^36^
30	USA	Wheat	Stem Elongation, Heading	Yield	NDVI	^37^
31	USA	Wheat	Stem Elongation, Heading	Yield, N uptake	NDVI	^43^
32	Denmark	Grass	GDD^4^ 432 - 861	PNC, Biomass	—	^63^
33	China	Rice	Tillering, Jointing, Booting	LNC	CIRE	^64^
34	China	Rice	Jointing, Booting, Heading	LNC	CIRE, CIREg, SAVI	^65^
35	USA	Switchgrass (*Panicum virgatum*)	End of season	PNC	NDRE	^66^
36	China	Rice	Jointing, Heading, Filling	Biomass	GOSAVI	^67^
37	China	Winter Wheat	Flowering - Ripening	NUE - Plant N Content	GNDVI, NDRE, RNDVI	^68^
38	China	Wheat	Stem Elongation, Heading, Flowering	Yield, N uptake, Biomass	DATT, RESAVI	^2^
39	China	Rice	Jointing, Booting, Heading, Filling	PNC	CIg, CIRE, NDVI, OSAVI, Viopt	^7^
40	China	Rice	Tillering, Jointing, Booting	LNA, PNA	CIRE, DATT, ENDVI, ExG, GNDVI, NGRDI	^69^
41	China	Rice	Jointing - Booting - Heading - Filling	PNC, LNC, LNA, PNA	NDRE	^70^

^1^Stages followed by a comma or dashes represent studies that assessed plant traits in each or across phenological stages, respectively. ^2^A missing vegetation index value indicates that only plant traits were assessed. Information regarding the full plant traits and VIs specifications are available on the figshare repository^39^. ^3^DAP: Days After Planting. ^4^GDD: Growing Degrees Days.

For each article presented in Table 1, all available information on VIs and plant traits from figures, tables, text, and supplementary material for figshare repository was extracted using the ‘juicr’ R package^35^. Also, the data were visually inspected to ensure the information was associated with plant development stage.

Among the 14 plant traits identified during data extraction, only two plant traits (i.e., relative yield and N uptake) could be combined, considering crop type (i.e., wheat and cotton), VIs (i.e., NDVI and NDRE), and phenological stage^3,15,33,34,36,37^.

### Data collection for meta-analysis

To explore the predictive abilities of drones in estimating agricultural traits, we undertook a meta-analysis encompassing 41 selected studies. This meta-analysis aimed to evaluate the potential of UAVs in estimating yield and nitrogen-related plant traits, with an approach that does not prioritize any VI. N-related traits (plant N density, plant N content, plant N concentration, plant N accumulation, NNI, N uptake, leaf N density, leaf N content, leaf N concentration, leaf N accumulation, canopy N content) were merged into a single category labeled “nitrogen” for simplification. This preprocessing step ensured consistency and clarity in trait categorization.

For each trait of interest (nitrogen and yield), we created individual plots. Within each plot, we iterated over growth stages and crops to calculate Fisher’s Z transformation effect sizes along with their 95% confidence intervals. This transformation converts the R² values into a metric that approximates a normal distribution, thereby making it more suitable for our analytic model. Fisher’s Z transformation was computed using Eq. (1):

1 $$Z=\frac{1}{2}ln\left(\frac{1+r}{1-r}\right)$$

where r represents the Pearson correlation coefficient, which was derived from the R² values provided in the dataset (supplementary material for figshare repository). The mean Fisher’s Z value and standard error were calculated for each group, and the error bars were plotted accordingly. Analysis was conducted separately for each growth stage and crop, facilitating comparative evaluations. When assessing the accuracy of plant trait estimations, we prioritize R² as our main metric due to its broad acceptance, straightforward interpretation, and most used metric compared to others.

Two types of regression analyses were performed to explore moderator effects: crop moderator analysis and growth stage moderator analysis. Ordinary Least Squares regression models were fitted to assess the influence of crop type and growth stage on R² values. One-hot encoding was applied to categorical crop variables and growth stages, with coefficients, standard errors, and p-values extracted to quantify the impact of individual crops and the role of different growth stages in trait prediction.

ANOVA was conducted to evaluate the significance of moderator effects, both for crop type and growth stage, on trait variability. F-values were computed for the entire sets of crops and growth stages, providing insights into the overall impact of these moderators on model fit. Data preprocessing, analysis, and visualization were performed using the Python programming language, leveraging libraries such as “Pandas”, “NumPy”, “Matplotlib”, and “Statsmodels”. These tools facilitated efficient data manipulation, statistical modeling, and graphical representation of results.

To standardize the data monitoring period across all studies, we converted the reported growth stages to the BBCH scale^38^, a very known scale for phenological staging. We categorized the growth stages as follows: early (BBCH 0–30), mid (BBCH 31–60), and late stage (BBCH 61–90). We also considered the entire growth period – all (BBCH 0–90) as a separate category. These categorizations were employed to assess the impact of different growth stages on the accuracy of N-related traits and yield prediction in major crops.

## Data Records

The data are accessible on the figshare repository^39^, available at 10.6084/m9.figshare.22938797, and includes the following files:

1. “Dataset.xlsx” includes the data. It contains three tabs: “UAV_dataset”, “Sensor and processing info”, and “Quantitatively analysis”.
2. “Summary of the dataset.docx”, includes a summary of the dataset excel file (UAV_dataset tab), defining each column, data extracted from the studies, the units for each variable when pertinent, and a definition for each variable.
3. “Figure2_N_Uptake.r”, includes the code to reproduce Fig. 2.

**Fig. 2:**
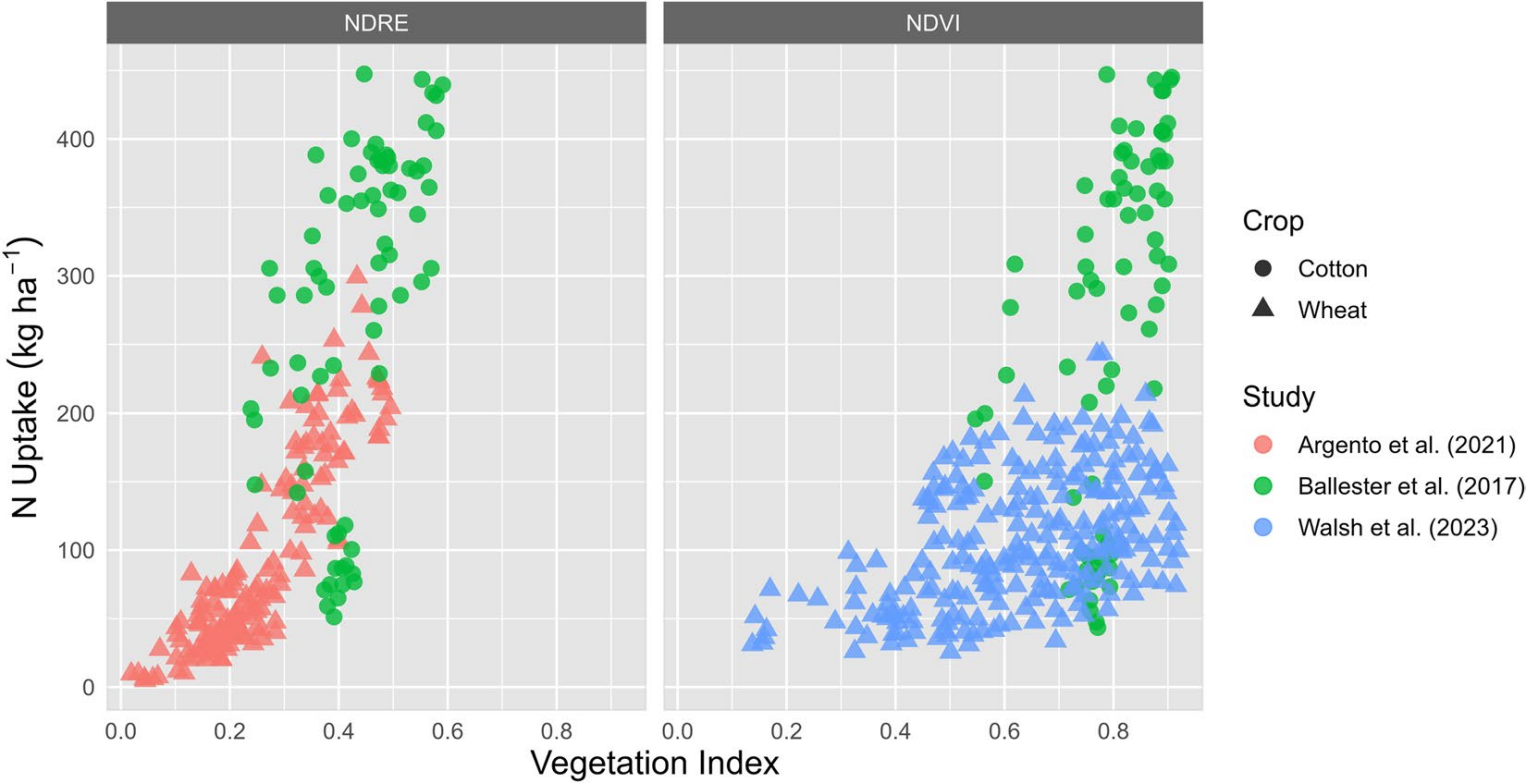
Relationship between nitrogen (N) uptake (kg ha^−1^) and two VIs (NDRE, NDVI) for cotton and wheat crops. Colors refer to different studies, and type of symbols represent the different field crop.
4. “Figure3_effect_size.ipynb”, includes the code to build Fig. 3.

**Fig. 3:**
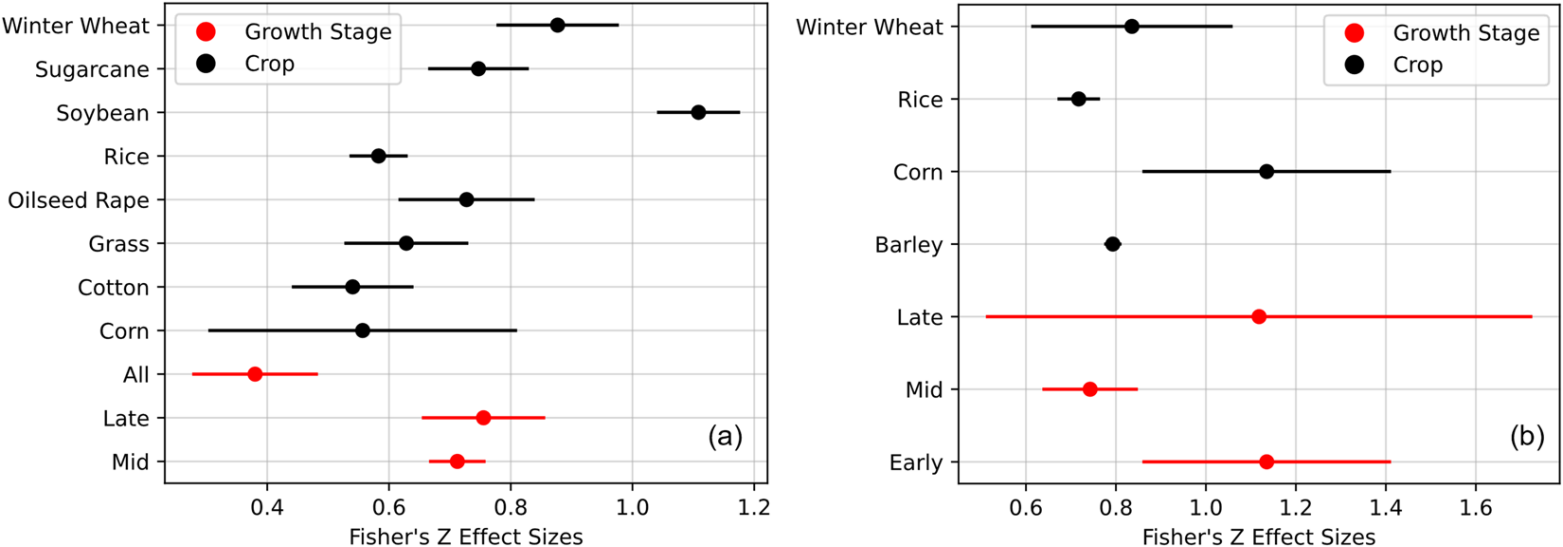
Observed Fisher’s *Z* effect sizes with their 95% confidence interval for nitrogen (**a**) and yield (**b**). Analysis across growth stages (red line) and crop varieties (black line).
5. “Figure4_RelativeYield_NDVI.r”, includes the code to run Fig. 4.

The “Dataset.xlsx” file (UAV_dataset tab) contains all the information collected on this systematic analysis. The “Summary of the dataset.docx” presents a description for each column of the “UAV_dataset” tab with the information separated into three categories:

Category I, general specification of the study, containing information for author and publication year, and paper identification for each study included in the dataset.

Category II, experiment information, describing species, VI used, VI value, coefficient of determination (R²), root mean square error (RMSE), and phenological stage sampling moment or dates.

Category III, key for the dataset related to plant traits used. All plant traits information is reported with their units, as expressed in the data collected from those respective studies. This category shows the amount of N rate applied, plant/leaf N concentration/content, N nutrition index, yield, relative yield, N uptake, leaf/plant N density, leaf/plant N accumulation, canopy N content, and the aboveground biomass values.

The “Dataset.xlsx” file (“Sensor and processing info” tab) describes topics related to sensor and processing information, including sensor band, spatial resolution, UAV flight height, plot size, VIs procedure, calibration of the sensor, weather/field condition, soil texture, latitude/longitude, and year of experiment for each of the 41 selected studies.

The “Dataset.xlsx” file (“Quantitatively analysis” tab) describes the study number, plant traits, R² metric, sample size for each trait (N), growth stage (BBCH scale), and field crop extracted. This tab was used for meta-analysis process.

Table 1 describes the main topics of the 41 selected studies, including species, country for the study location, author, and year of publication, phenological stage sampling moment, plant traits and VIs utilized for each study, and relevant keyword for the study.

Table 2 describes the regression models with one moderator (crop or growth stage) for nitrogen and yield plant traits. This table investigates the predictive capabilities of drones in estimating agricultural traits without focusing on specific VI.

**Table 2:** Regression models with one moderator (Crop, Growth Stage) for Nitrogen and Yield plant traits.

Moderator	Regression Model Statistics			Anova	Regression Model Statistics			Anova
	Nitrogen			F	Yield			F
	Esti.	SE	p-value		Esti.	SE	p-value	
**Crop**				4.8				0.9
Barley	—	—	—		0.660	0.005	0.000**	
Corn	0.487	0.096	0.015*		0.754	0.064	0.000**	
Cotton	0.461	0.036	0.000**		—	—	—	
Grass	0.514	0.033	0.000**		—	—	—	
Oilseed Rape	0.605	0.036	0.000**		—	—	—	
Rice	0.501	0.018	0.000**		0.615	0.015	0.015*	
Soybean	0.802	0.012	0.000**		—	—	—	
Sugarcane	0.630	0.025	0.000**		—	—	—	
Winter Wheat	0.637	0.029	0.000**		0.626	0.061	0.000**	
**Growth Stage**				16.98				1.83
Early	—	—	—		0.754	0.064	0.000**	
Mid	0.568	0.014	0.000**		0.613	0.034	0.000**	
Late	0.585	0.030	0.000**		0.717	0.166	0.023*	
All	0.344	0.047	0.000**		—	—	—	
N° of studies: 25					N° of studies: 11			

Esti.: Estimated Coefficient; SE: Standard Error; p-value; F: F-statistic; - No data available for some crops and growth stage depending on the trait assessed. Significance levels: **p < 0.001, *p < 0.05.

## Technical Validation

To demonstrate the value of the dataset, the relationship between VIs and plant traits was investigated. After constructing the dataset, we checked for potential outliers and carefully summarized the information to analyze the interaction of multiple studies with the goal of merging them.

Data of N uptake and two VIs (NDRE and NDVI) are presented in Fig. 2 for three studies across stages and crops (cotton and wheat). These three studies were conducted in Switzerland, Australia, and the United States for wheat crop at three stages (tillering, stem elongation, and heading) and then for cotton crop at three stages (first flower, first cracked boll, and maturity). The N uptake (kg N ha^−1^) was calculated by multiplying the dry matter biomass (kg ha^−1^) with the corresponding N concentration (%) of the plant sample^40^. Although these two crops are managed differently with respect to crop management such as time of nutrient application, the overall trend was similar between VIs and the key plant N trait identified for these crop species.

In contrast to NDRE, NDVI saturated shortly after stem elongation before decreasing rapidly during the senescence phase1 when studies were combined (saturation point when NDVI ≥ 0.5). The NDVI seems to be a viable N status indicator for a first N application, when the crop leaf canopy has not closed yet. The NDRE progress was linear until the stage of spike emergence, which takes place after the fertilizer application in winter wheat. Thus, it is plausible that NDRE is a better VI for the creation of fertilizer prescription maps and N uptake assessment than NDVI. Moreover, the correlation value for NDRE (R^2^ = 0.80 and 0.67 for wheat and cotton, respectively) confirmed the assumption that NDRE can be used to monitor the N status of the wheat and cotton crops. Lastly, these studies provide initial evidence of the potential superiority of red-edge-NIR based spectral indices over NDVI (R^2^ between 0.11–0.65 across crops)^40,41^. In addition, this dataset helps to demonstrate the need of expanding the exploration of other spectral bands to target specific plant N traits more directly.

Data collected can also be used to predict N-related traits and yield for the given field crop and growth stage, merging all the VIs used in each paper. For N-related traits, the F-value for the entire crop set and growth stage was 4.91 and 16.79, respectively (Table 2). The F-values for the entire growth stage set are notably higher than those for the entire crop set, suggesting that growth stage may have a stronger influence on these traits compared to crop type. However, for yield, both the F-values for entire crop sets and entire growth stage sets are relatively low, indicating that neither crop type nor growth stage may have a significant impact on yield in this dataset.

The coefficients and associated p-values indicate that different crops have significant effects on N content and yield. For instance, crops like cotton, grass, oilseed rape, rice, soybean, and winter wheat show significant effects on N-related traits, with p-values indicating a positive relationship between these crops and higher R² values for N-related vegetation indices. Similarly, barley, corn, rice, and winter wheat show significant positive coefficients (p < 0.05), indicating their positive influence on R² values for yield-related vegetation indices, highlighting their suitability for accurate yield prediction using remote sensing data.

Notably, the mid and late growth stages further enhanced the model predictive capability for the N trait estimation, indicating their positive influence on R² values for N-related vegetation indices derived during these stages. Compared with all growth stage (Estimate = 0.344, SE = 0.047, p < 0.001), both mid stage (Estimate = 0.568, SE = 0.014, p < 0.001) and late stage (Estimate = 0.585, SE = 0.030, p < 0.001) showed a more significant influence, reflecting the relevance of aiming for a specific growth stage to estimate N during the plant development (Table 2). While our dataset lacked early growth stage data based on the BBCH scale, the absence of this information underscores the importance of early-stage data. This indicates that the initial assimilation of N strongly influences subsequent plant productivity^2,37,42^. In the context of yield, it was unexpected to observe that the early stage showed a high estimate accuracy (Estimate = 0.754, SE = 0.064, p < 0.001), followed by the late stage (Estimate = 0.717, SE = 0.166, p < 0.001) with a higher standard error. The higher estimation accuracy for yield at the early growth stages may be attributed to the distinct spectral signatures captured by VIs where unique phenological signatures are assessed, indicating rapid vegetative growth or early stressors, leading to higher accuracy in yield estimation.

Results also reveal the uncertainty (reflected as the length of the 95% credibility interval) is higher for the late growth stage yield prediction (Fig. 3). The level of uncertainty depends on the number of observations within a study and on the total number of studies for a growth stage. We found only three studies that used the late stage to estimate yield^2,15,32^. When the number of data is small, the determination of yield can produce estimates with large uncertainty (wide credibility intervals).

## Usage Notes

**Fig. 4:**
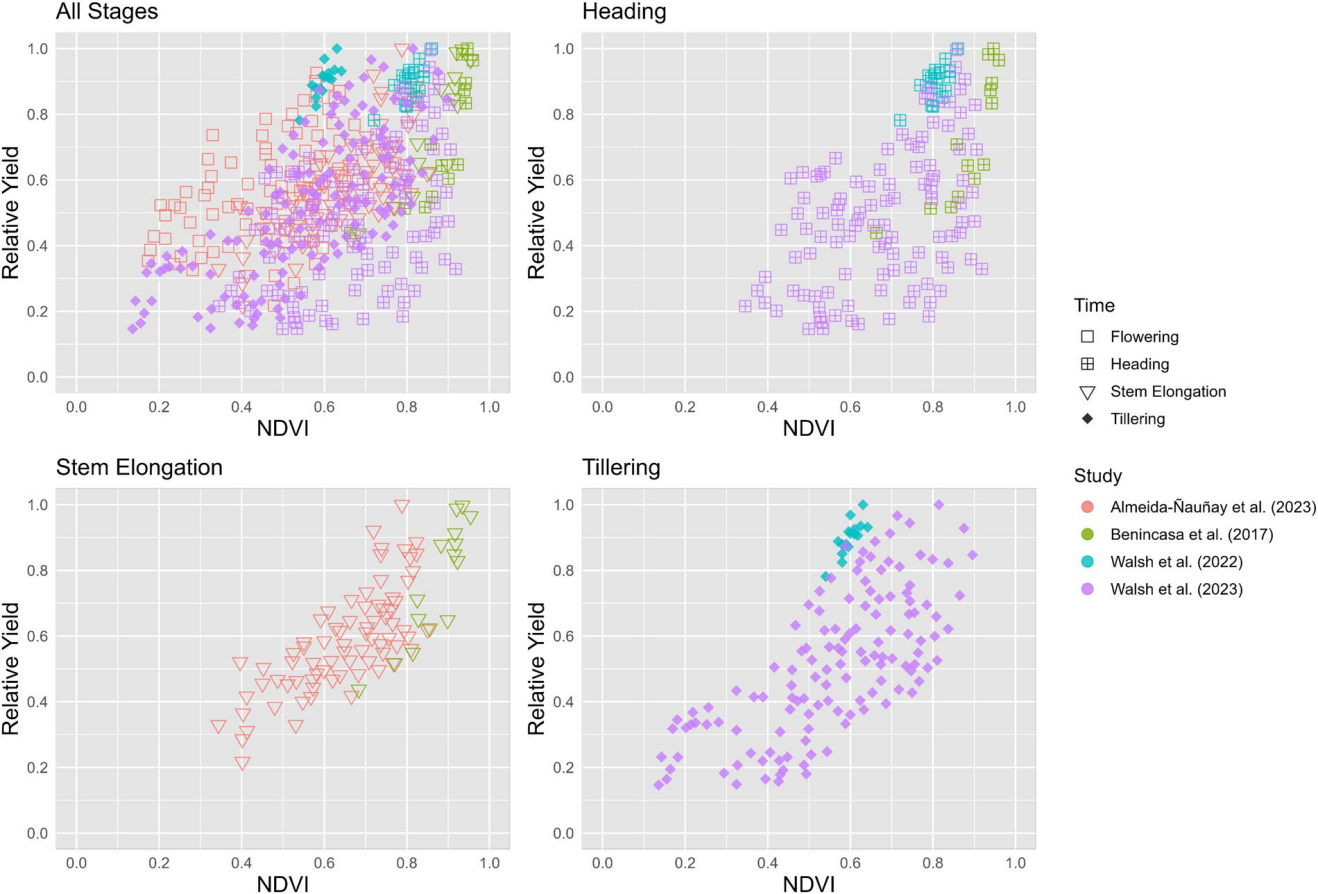
Relationship between relative yield and NDVI for wheat comprising all stages and individual ones (tillering, stem elongation and heading) combined. Colors represent different studies, and the type of symbols refer to the crop growth stage (time).

This dataset can also be used in studies to diagnose N status and various plant traits in different crop species using UAV imagery. For example, recent studies have used NDVI as a yield predictor for wheat^3,15,37,43^. However, when combining data from these studies conducted across different environments, it is not possible to gain insights about the relationship between relative yield and NDVI (Fig. 4). It is noteworthy to understand that different growth stages will present varying conditions, which indicates the need to properly report crop phenology (growth stage) and environmental conditions (rainfed vs. irrigated) when correlating yield with any VI^44^. For instance, some studies have highlighted the importance of obtaining an estimation of crop biomass in reducing variability/noise when exploring crop N status^45^. This approach could lead to more reliable models and the development of more universal N management guidelines.

Despite considerable progress, there are still many relevant research knowledge gaps in drone-based crop research. Many studies do not use the same VIs to analyze a specific plant trait and/or phenological stage. In addition, numerous studies provided metrics (R² and/or RMSE) of the relationship between plant trait and VI, but often omitted the corresponding data, restricting the future use of those studies.

Additionally, the dataset could be expanded to include other plant traits such as other nutrient deficiencies (e.g., potassium), drought status, and pest and disease detection. Drone-based imagery data can help detect changes in crop N status early in the season, permitting to adjust via interventions. Improving the ability to more precisely and dynamically correct crop N deficiencies will help farmers focus on a more sustainable approach to monitor large areas in a short period of time, improving farming profitability and reducing the environmental footprint.

## Data Availability

Scripts using R and python programming languages are provided to produce figures. Additional code and related files are available at figshare repository^39^.
